# The Optimal Timing of Enterostomy Closure in Extremely Low Birth Weight Patients for Acute Abdomen

**DOI:** 10.1038/s41598-018-33351-9

**Published:** 2018-10-24

**Authors:** Hee-Beom Yang, Ji-Won Han, Joong Kee Youn, Chaeyoun Oh, Hyun-Young Kim, Sung Eun Jung

**Affiliations:** 1 Department of Pediatric Surgery, Seoul National University Children’s Hospital, Seoul, Korea; 2 Department of Pediatric Surgery, Seoul National University, College of Medicine, Seoul, Korea

## Abstract

There are few reports on enterostomy closure (EC) timing for acute abdomen in extremely low birth weight (ELBW) patients. We retrospectively reviewed ELBW patients who underwent enterostomy formation (EF) and subsequent EC. We investigated baseline characteristics, surgical outcomes, and follow-up data of 55 patients and analyzed optimal timing by age at EC, enterostomy duration, and body weight (Bwt) at EC. The minimum p-value approach (MPA) using the Chi-squared test was used to determine each cut-off value. Mean gestational age was 25^+3^ weeks, while mean age and Bwt at EF were 10 days and 660 g. Enterostomy duration and Bwt at EC were 102 days and 2400 g. Fourteen surgical complications were related to EC. The MPA identified a cut-off of 2100 g (*p* = 0.039) at EC but no significant cut-off age or enterostomy duration. The 18 patients <2100 g had more enterostomy-related problems at EC than the >2100 g group (66.7% vs 10.8%, *p* < 0.001). No other characteristics were significantly different. Operation time, ventilator period, hospital stay, parenteral nutrition duration, and full feeding day were significantly longer in <2100 g patients. Follow-up Bwt did not differ (11.55 kg vs 13.95 kg, *p* = 0.324). Our findings suggest EC can be safely performed when Bwt is over 2100 g.

## Introduction

The development of perinatal management has made it possible for low birth weight (LBW) infants to survive. Accordingly, abdominal operations have increased due to an increased incidence of acute abdomen conditions such as necrotizing enterocolitis (NEC) and spontaneous intestinal perforation (SIP) in these babies^1,2^. Treatment in cases of acute abdomen can be medical or surgical according to disease severity or patient condition. Surgical options include primary closure, primary anastomosis with intestinal resection, enterostomy with or without intestinal resection, and peritoneal drainage. Although controversy exists about what is the best treatment for each patient, enterostomy is a generally accepted safe treatment option for LBW patients with acute abdomen^3–6^.

There are a few studies of the timing of enterostomy closure (EC), but patients, variables, and criteria of each study for identifying the optimal timing of EC are diverse. Lee J. *et al*. showed body weight (Bwt) at EC is a significant factor for the development of complications after EC in patients with NEC^7^, while Kang M. *et al*. showed a similar result using the corrected age at EC for preterm patients with NEC^8^. Other reports used enterostomy duration to determine timing, but the criteria (4–10 weeks) and patient groups (NEC or acute abdomen patients) varied^9–11^.

**Figure 1:**
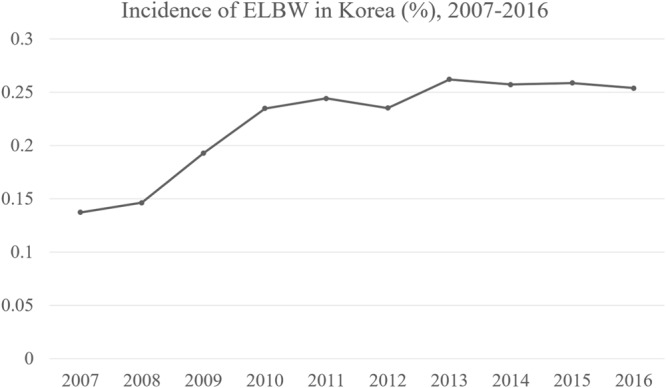
Incidence of extremely low birth weight (ELBW) infants in Korea (total ELBW newborns/total newborns yearly), 2007–2016, raw data from Statistics Korea.

The incidence of extremely LBW (ELBW) is increasing in Korea (Fig. 1). Some studies have included preterm or LBW patients, but few reports have included only ELBW patients^7,10–16^. Thus, it is undetermined whether previously published are applicable to ELBW patients, so we conducted this study to investigate the optimal timing of EC in ELBW patients with acute abdomen.

## Methods

This retrospective single center study was conducted at Seoul National University Children’s Hospital in South Korea. We reviewed the data of ELBW infants who underwent laparotomy for acute abdomen, including NEC, SIP, meconium-related ileus (MRI), and meconium non-related ileus (MNRI) between January 2003 and April 2016. Children who weighed >1000 g at the time of the first ileostomy formation were excluded. Patients who did not undergo EC were also excluded. Patients’ characteristics, mode of delivery, cause of preterm birth, multiple pregnancy, Apgar score, preterm complications, enterostomy formation (EF)- and closure-related characteristics, EC outcomes, and follow-up data were investigated. This study was approved by the institutional review board of Seoul National University Hospital. We were waived the informed consent by the institutional review board because of the nature of this retrospective study. All methods used in this study were performed in accordance with the relevant guidelines and regulations.

The clinical criteria for closing enterostomy in our institution is body weight for patients otherwise stable without high output enterstomy or prolapse. 2.0 kg of body weight was considered as safe for performing enterostomy closure. Other procedures such as drainage or segmental resection were considered for SIP, but enterostomy formation was procedure of choice in our institution for SIP occurring in ELBW infants.

Acute abdomen was classified into four categories according to the intraoperative findings and pathologic results: NEC, SIP, MRI, or MNRI. Midgut volvulus and congenital anomaly such as intestinal atresia were not considered to enroll in this study. NEC is defined as necrosis of the mucosal and submucosal layers of the bowel, while SIP is an isolated perforation of the bowel with no obvious inflammation in the rest of the bowel^17,18^. MRI is characterized by a functional ileus caused by impaired meconium excretion not associated with cystic fibrosis and is usually diagnosed with a dilated proximal ileum filled with sticky meconium^19^, whereas MNRI is an ileus without a definite sticky meconium.

Z-score is the number of standard deviations from the mean of the general population. The World Health Organization AnthroPlus program was used to calculate z-score of Bwt and height for postmenstrual age >40-week patients and PediTools (http://peditools.org/fenton2013) for <40-week patients. Total parenteral nutrition (TPN)–related cholestasis was defined as a direct bilirubin level >2 mg/dL with TPN use. Patients with an already elevated direct bilirubin level before TPN use were excluded. In cases of double-barrel enterostomy, the proximal and distal bowel may differ. Enterostomy type was defined as proximal bowel regardless of distal bowel. Three routine blood tests (white blood cells, hemoglobin [Hb], and albumin) performed within 2 weeks prior to the operation were used to assess the nutritional status of ELBW patients at the time of EC. The full enteral feeding day was defined as the day the patient reached a feeding amount of 100 mL/kg/day. Follow-up Bwt is the last Bwt measurement at the outpatient department (OPD).

The minimum *p*-value approach (MPA) was used to determine the optimal cut-off for several variables including age, corrected age, Bwt at EC, and enterostomy duration. Chi-squared tests were used to test for significant intergroup differences (low vs high) for EC-related complications at each threshold. The optimal cut-off was chosen as the value that yielded the maximum Chi-squared or minimal *p*-value on the Chi-squared test. The *p*-value for the optimal cut-off point by MPA was corrected using the two-fold cross-validation technique to control type I errors.

SPSS version 23 (IBM Corporation, Armonk, NY, USA) was used for the statistical analysis. All continuous variables are presented as median values and range. The Mann-Whitney *U* test was used to examine continuous data, while the Chi-squared test was used to analyze categorical data. Multivariable logistic regression was performed to adjust for confounding covariates and to examine the result of MPA method on the complication, which included covariates of p-value < 0.05 in difference between groups. The comparison between pre-closure and follow-up Bwt z-score was performed using a paired t-test. *P*-values < 0.05 were considered statistically significant.

## Results

**Figure 2:**
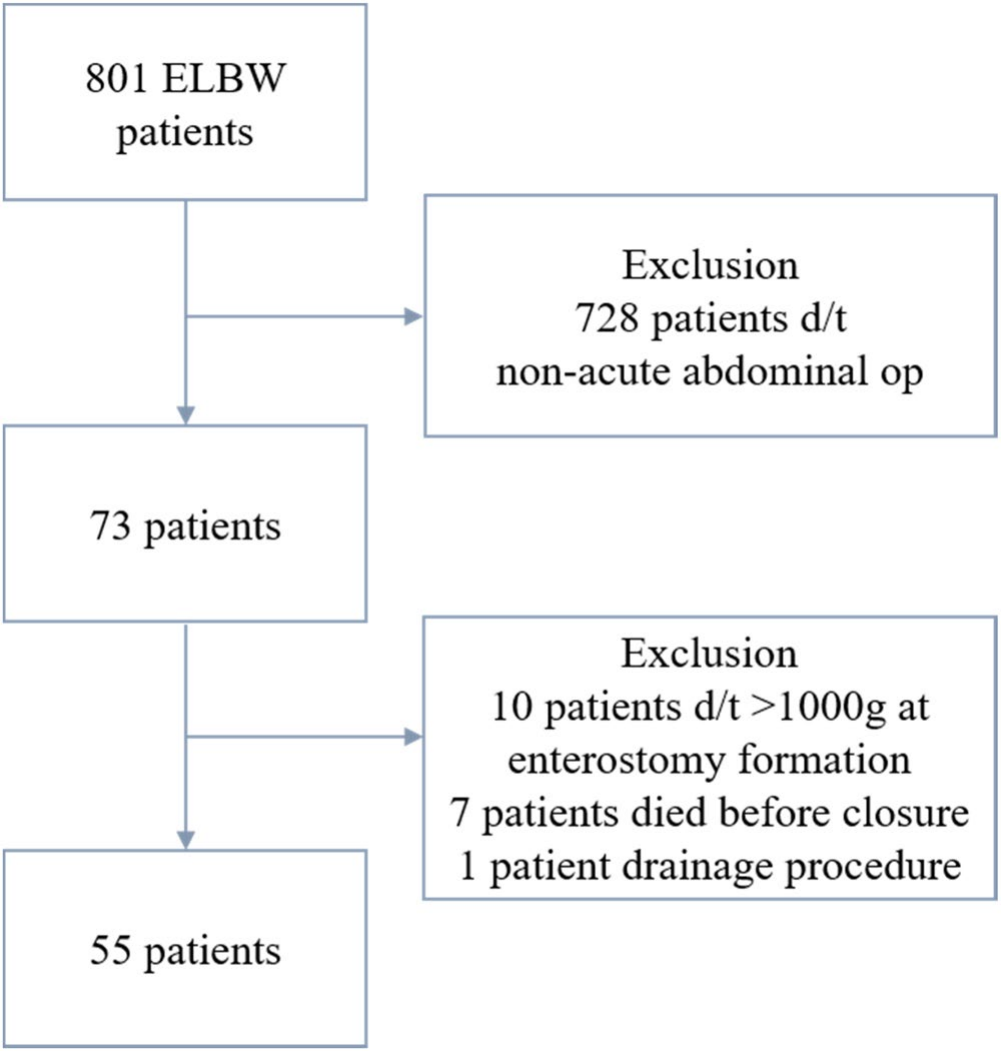
Flow chart of patient inclusion process.

A total of 801 ELBW infants were admitted during study duration; of them, 728 who did not undergo laparotomy for acute abdomen were excluded, 10 were excluded due to not meeting Bwt criteria at EC (<1000 g), seven died before the EC, and one who underwent peritoneal drainage was also excluded. Thus, a total of 55 ELBW patients were analyzed in this study (Fig. 2). The characteristics of 7 patients who died before EC were shown in Table 1.

**Table 1:** Patients who underwent enterostomy and died before enterostomy closure.

Case	Sex	GA (week + day)	Birth weight (g)	Age at op (day)	Body weight at op (g)	Dx	Op	Cause of death	Periods from op to death (day)
1	F	25 + 1	560	24	570	NEC	Jejunostomy	Sepsis	1
2	M	23 + 1	540	10	580	NEC	Ileostomy	Intraop cardiac arrest	0
3	F	22 + 3	480	17	490	NEC	Ileostomy	Adrenal insufficiency	14
4	M	27 + 2	610	34	870	NEC	SB resection and ileostomy	Respiratory failure due to BPD	128
5	M	30 + 4	770	9	850	MRI	Jejunostomy and ileostomy	Sepsis	28
6	M	27 + 1	670	15	700	NEC	SB resection and jejunostomy	Sepsis	48
7	F	23 + 2	440	29	640	SIP	Jejunostomy	Sepsis	21

GA: Gestational age, Op: Operation, Dx: Diagnosis, F: Female, M: Male, NEC: Necrotizing enterocolitis, MRI: Meconium related ileus, SIP: Spontaneous intestinal perforation, BPD: Bronchopulmonary dysplasia.

### Patient characteristics and outcomes

Thirty-seven of our patients were male. Median GA was 25^+3^ weeks (range, 23^+1^ to 32^+1^ weeks). Median birth weight was 710 g (range, 430–990 g). A total of 19, 17, 14, and five patients had NEC, SIP, MRI, and MNRI, respectively. A total of 37 patients were born by Cesarean section (C/S). Causes of preterm birth included premature rupture of membrane in 17 cases, spontaneous labor in 12 cases, and labor induction or C/S for fetal (n = 17) or maternal (n = 9) indications. The other characteristics are shown in Table 2.

**Table 2:** Patients’ characteristics.

	N = 55
Male	37
Gestational age (w)	25 + 3 (23 + 1–32 + 1)
Birth weight (g)	710 (430–990)
z-score	−0.49
Small for gestational age	20
Diagnosis	
NEC	19
SIP	17
MRI	14
MNRI	5
Mode of delivery	
Cesarean section (C/S)	37
Vaginal delivery	18
Cause of preterm birth	
PPROM	17
Spontaneous labor	12
Labor induction or C/S	
Fetal indications	17
Maternal indications	9
Multiple pregnancy	
No	28
Twin	22
Triple	5
Apgar score	
1 min	2 (0–7)
5 min	5 (0–8)
Preterm complications	
RDS	45
PDA	50
Ligation operation	25
ROP	32
Brain lesion	
Hemorrhage	29
PVE	19
Normal	7
Mechanical ventilator after birth (d)	31 (0–385)
Maternal age at delivery (y)	32 (24–43)

NEC: necrotizing enterocolitis, SIP: spontaneous intestinal perforation, MRI: meconium-related ileus, MNRI: meconium non-related ileus, PPROM: preterm premature rupture of membrane, RDS: respiratory distress syndrome, PDA: patent ductus arteriosus, ROP: retinopathy of prematurity, PVE: periventricular enlargement.

Median age at EF was 10 days (range, 2–43 days). Median Bwt at EF was 660 g (range, 430–940 g). A total of 19 patients had TPN-related cholestasis before EC. 15 patients underwent 2 or more abdominal operations before EC. A total of 47 patients underwent ileostomy, while eight underwent jejunostomy. Median age at EC was 117 days (range, 65–984 days). Median enterostomy duration was 102 days (range, 38–965 days). Median Bwt at EC was 2400 g (range, 1370–12050 g). Sixteen patients had stoma-related problems at EC. The other EF and EC characteristics are shown in Table 3.

**Table 3:** Clinical characteristics with enterostomy formation and closure.

	N = 55
Enterostomy formation	
Age (d)	10 (2–43)
Body weight (g)	660 (430–940)
TPN-related cholestasis before EC	
Yes	19
No	32
Unknown	4
Multiple abdominal op before EC	15^*^
PN stopped before EC	51
Enterostomy refeeding	10
Type of enterostomy	
Ileostomy	47
Jejunostomy	8
Enterostomy closure	
Age (d)	117 (65–984)
Corrected age (d)	15 (−32–882)
Enterostomy duration (d)	102 (38–965)
Body weight (g)	2400 (1370–12050)
z-score	−2.86 (−5.72–0.02)
Height (cm)	44.0 (38–87.6)
z-score	−3.49 (−8.39–0.6)
Enterostomy-related problem at closure	16^†^
Same hospitalization with EF	36
Lab	
WBC (10^3^/μL)	10.27 (5.03–22.25)
Hb (g/dL)	11.1 (8.0–14.5)
Albumin (g/dL)	3.7 (2.4–4.6)

EF: enterostomy formation, EC: enterostomy closure, TPN: total parenteral nutrition, PN: parenteral nutrition, WBC: white blood cell, Hb: hemoglobin.

^*^Multiple abdominal op: 7 for complications after enterostomy, 3 for correction after primary closure or anastomosis, 1 for definite op after drainage procedure, 1 for 2^nd^ look op, 1 for intestinal biopsy d/t persistent delayed evacuation for contrast study, 1 for perforation during contrast study, 1 for transformation from jejunostomy to ileostomy.

^†^Enterostomy -related problem: 6 for high output enterostomy, 3 for enterostomy prolapse, 3 for failure to thrive, 1 for both high output and prolapse, 1 for accidental, 1 for peristoma skin problem, 1 for parents’ require.

Median operation time was 75 minutes (range, 25–155 minutes). Twelve patients received a transfusion at EC. Median hospital stay, EN start day, and full enteral feeding days were 18, 6.5, and 9 days, respectively. A total of 14 complications occurred: ileus (n = 7), wound complications (n = 3), incisional hernia (n = 3), and fluid collection identified with sonography (n = 1). Three cases of incisional hernia and two cases of ileus required reoperation. The median follow-up period was 1883 days (range, 42–4992 days). The median follow-up Bwt was 12.7 kg (range, 2.7–25.6 kg). Two deaths occurred. The other outcome and follow-up data are shown in Table 4.

**Table 4:** Outcome of enterostomy closure.

	N = 55
Enterostomy closure outcome	
Op time	75 (25–155)
EBL	0 (0–130)
Intraop transfusion	12
Transfusion within 3 days	29
Mechanical ventilator (d)	0 (0–77)
Hospital stay (d)	18 (6–345)
PN duration (d)	8 (0–38)
EN start (d)	6.5 (4–26)
Full enteral feeding day	9 (5–37)
Complication	14
Postop ileus	7
Wound complication	3
Incisional hernia	3
Fluid collection	1
Reoperation	5
Incisional hernia	3
Postop ileus	2
Follow up	
Follow up period (d)	1883 (42–4992)
Corrected age at anthropometric measurement (d)	1247 (42–3649)
Body weight (kg)	12.7 (2.7–25.6)
z-score	−1.52 (−4.88–0.84)
Height (cm)	95.05 (47.3–134.1)
z-score	−1.16 (−6.82–1.52)
Death	2

EBL: estimated blood loss, PN: parenteral nutrition, EN: enteral nutrition.

### MPA analysis

**Figure 3:**
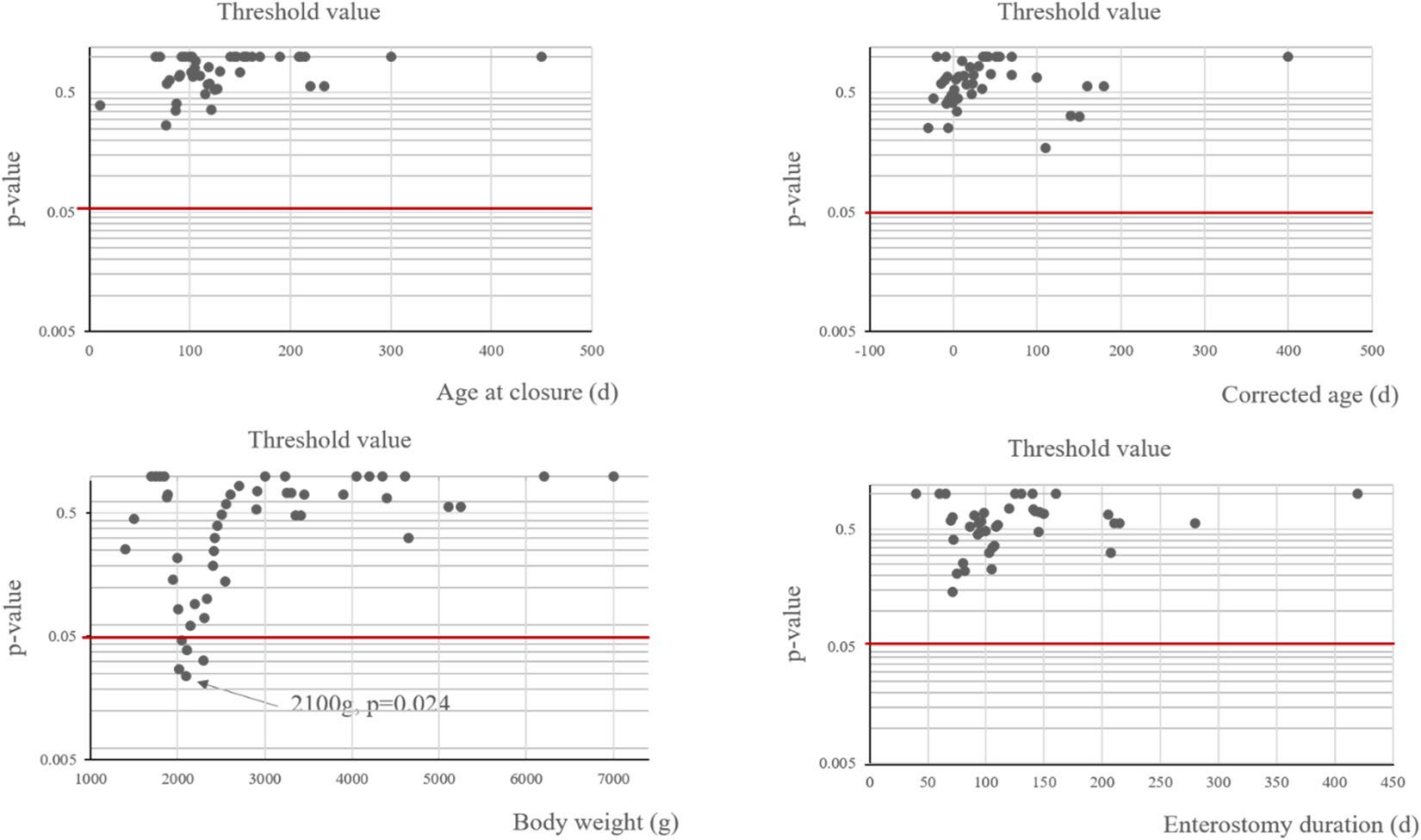
Results of minimum p-value analysis with respect to age, corrected age, body weight at enterostomy closure (EC), and enterostomy duration for EC-related complication. Only body weight at EC showed significant cut-off values. Among them, 2100 g yielded the minimum *p*-value in the Chi-squared test.

We analyzed the optimal timing of EC with respect to Bwt at EC, age at EC, corrected age at EC, and enterostomy duration. As previously explained, we performed MPA using the Chi-squared test (Fig. 3).

There were no significant cut-off values for age at EC, corrected age at EC, or enterostomy duration. In fact, there was a significant cut-off value only in Bwt at EC. Among several values showing significance, a Bwt of 2100 g was the value showing minimum *p*-value (*p* = 0.024). That is, the complication rate was different most significantly at the value of 2100 g at EC. This result was still significant after use of the two-fold cross-validation technique to control type I error (*p* = 0.039).

### Comparison of patients according to MPA analysis

We divided the patients into two groups with the cut-off value of 2100 g. Group 1 (n = 18) included patients with a Bwt < 2100 g at EC, while group 2 (n = 37) included those with a Bwt > 2100 g at EC. Detailed comparisons are shown in Table 5.

**Table 5:** Comparison according to body weight.

	<2100 g (N = 18)	≥2100 g (N = 37)	*p*-value
Patients’ characteristics			
Male	12 (66.7%)	25 (67.6%)	0.947
Gestational age (w)	27 + 0	25 + 3	0.07
Birth weight (g)	650	710	0.212
z-score	−1.17	−0.36	0.074
Small for gestational age	9 (50.0%)	11 (29.7%)	0.143
Diagnosis			0.884
NEC	7 (38.9%)	12 (32.4%)	
SIP	5 (27.8%)	12 (32.4%)	
MRI	5 (27.8%)	9 (24.3%)	
MNRI	1 (5.6%)	4 (10.8%)	
C/S	15 (83.3%)	22 (59.4%)	0.077
Cause of preterm birth			0.130
Maternal factor	10 (55.6%)	28 (75.7%)	
Fetal factor	8 (44.4%)	9 (24.3%)	
Multiple pregnancy	8 (44.4%)	19 (51.4%)	0.631
Apgar score			
1 min	3	2	0.222
5 min	6	5	0.151
RDS	14 (77.8%)	31 (83.8%)	0.713
PDA operation	7 (38.9%)	18 (48.6%)	0.495
ROP	9 (50.0%)	23 (62.2%)	0.391
Brain hemorrhage	10 (55.6%)	19 (51.4%)	0.769
Mechanical ventilator after birth (d)	10.5	39	0.175
Maternal age at delivery (y)	33	31	0.076
Enterostomy formation			
Age (d)	8.5	10	0.699
Body weight (g)	660	700	0.740
Multiple op before closure	6 (33.3%)	9 (24.3%)	0.481
PN stopped before closure	15 (83.3%)	36 (97.3%)	0.061
TPN related cholestasis	7 (41.2%)	12 (35.3%)	0.682
Enterostomy refeeding	2 (11.1%)	8 (21.6%)	0.470
Type of enterostomy			0.416
Ileostomy	14 (77.8%)	33 (89.2%)	
Jejunostomy	4 (22.2%)	4 (10.8%)	
Enterostomy closure			
Age (d)	96	136	<0.001
Corrected age (d)	−4	34	<0.001
Enterostomy duration (d)	76.5	123	<0.001
Body weight (g)	1875	2920	<0.001
z-score	−3.49	−2.61	0.002
Height (cm)	40.75	46.8	<0.001
z-score	−4.38	−2.94	0.014
Enterostomy-related problem at closure	12 (66.7%)	4 (10.8%)	<0.001
Same hospitalization with EF	18 (100%)	18 (48.6%)	<0.001
Lab			
WBC (10^3^/μL)	11.51	10.12	0.101
Hb (g/dL)	10.1	11.4	0.006
Albumin (g/dL)	3.3	3.8	0.002
Enterostomy closure outcome			
Op time (min)	88	69	0.028
EBL (cc)	0	0	0.809
Intraop transfusion	6 (33.3%)	6 (16.2%)	0.149
Transfusion within 3 days	13 (72.2%)	16 (43.2%)	0.043
Ventilator period	1	0	0.048
Hospital stay	26.5	13	<0.001
PN duration	12.5	7	<0.001
EN start day	7.5	6	0.02
Full feeding day	11.5	9	0.007
Complication	8 (44.4%)	6 (16.2%)	0.024
Reoperation	3 (16.7%)	2 (5.4%)	0.317
Follow up			
Follow up period (d)	1919	1796	0.733
Corrected age at anthropometric measurement (d)	1439	1144	0.774
Body weight (kg)	11.55	13.95	0.324
z-score	−1.76	−1.27	0.132
Height (cm)	94.25	95.25	0.582
z-score	−1.28	−0.98	0.179
Death	0	2	>0.99

NEC: necrotizing enterocolitis, SIP: spontaneous intestinal perforation, MRI: meconium-related ileus, MNRI: meconium non-related ileus, RDS: respiratory distress syndrome, PDA: patent ductus arteriosus, ROP: retinopathy of prematurity, PN: parenteral nutrition, TPN: total parenteral nutrition, EF: enterostomy formation, WBC: white blood cell, Hb: hemoglobin, EBL: estimated blood loss, EN: enteral nutrition.

There were no significant differences in any demographic variables except enterostomy-related problems at EC (66.7% in group 1 vs 10.8% in group 2) (*p* < 0.001).

As shown above the MPA, *p*-value for complication was 0.024. Other EC outcomes also showed significant intergroup differences. Operation time, mechanical ventilator period, and hospital stay after EC were significantly longer in group 1 (p = 0.028, 0.048, <0.001, respectively). Nutritional factors like PN duration and full feeding day also showed significant differences. More patients in group 1 than in group 2 required transfusion within 3 days after EC (72.2% vs 43.2%, *p* = 0.043).

The follow-up period did not differ significantly between groups (1919 days vs 1796 days, *p* = 0.733). Median Bwt and z-score were slightly lower in group 1 than in group 2 (11.55 vs 13.95 kg and −1.76 vs −1.27, respectively), but the difference was not statistically significant (*p* = 0.324, 0.132).

### Multivariable Analysis

Logistic regression test was performed with covariates in Table 5 excluding EC outcome and follow up variables. Age, enterostomy duration, enterostomy-related problems at closure were excluded for covariates due to high correlation with body weight at EC. Body weight group according to MPA method, Hb, and albumin at EC were included. After adjusting potential confounding factors, body weight group was still statistically significant (odds ratios (OR) = 5.124, 95% confidence interval (CI) 1.076–24.390, *p* = 0.040) (Table 6).

**Table 6:** Multivariable Analysis.

	Odds ratio	95% confidence interval	*p*-value
Body weight group (<2100 g, >2100 g)	5.124	1.076–24.390	0.04
Hb	1.394	0.857–2.269	0.180
Albumin	0.545	0.112–2.643	0.451

## Discussion

Several studies have reported on EC timing in various patient groups. However, it is difficult to apply those results to ELBW patients due to the wide distribution of patient age and Bwt. The present study included only ELBW infants weighing <1000 g who underwent EF to control patients’ characteristics. Thus, this report may help clinicians decide the timing of EC in ELBW patients.

Although many studies have examined EC timing, it is still difficult to make a definite conclusion about when to perform EC. One report analyzed 89 patients who were <6 months of age at the time of EC^13^. The patients were stratified into four groups based on their Bwt at EC (<2 kg, 2–2.5 kg, 2.5–3.5 kg, and ≥3.5 kg). There was no significant intergroup difference in major morbidities, and only incisional hernia was significantly different in the <2 kg group. However, in that study, patients were arbitrarily stratified by age as older or younger than 6 months. Veenstra M *et al*. analyzed the patients who underwent EC for NEC by dividing them into enterostomy duration of 8 weeks, 8–12 weeks, or 12 weeks^16^. They reported no significant differences in PN duration, ventilator period, complication, and mortality. However, Banerjee DB *et al*. reported that NEC patients who underwent earlier EC with an enterostomy duration <10 weeks had a higher morbidity rate than those who underwent EC later with an enterostomy duration >10 weeks^10^. Bethell G *et al*. divided patients according to complications or no complications^14^ and reported that 24 patients with complications weighed less than 34 patients without complications (3655 g vs 5185 g, respectively). Older for GA and older corrected age at EF was seen in the no complications group (35 weeks vs 28.5 weeks, *p* = 0.01; and 36.3 weeks vs 32.6 weeks, *p* = 0.011).

In our study, we found no significant age-based factors related to complications. In contrast, a study of patients who were <37 weeks of GA in 2013^8^ showed that corrected age was a significant factor for complications. They divided patients into groups younger than or older than 40 weeks. The method differed from that of our study, making comparisons difficult; however, we found it interesting that there was a significant difference in complications by age.

The median values for the characteristics of our patients are as follows: GA, 25^+3^ weeks; birth weight, 710 g; age at EF, 10 days; Bwt at EF, 660 g; age at EC, 117 days; Bwt at EC, 2400 g; and postmenstrual age at EC, 42 weeks. The postoperative hospital stay was 18 days; 25.5% of the patients had complications. At the time of OPD, the patients were a mean 3.4 years of age, had a mean height of 95.5 cm, and had a mean weight of 12.7 kg (z-scores, −1.16 and −1.52, respectively). The z-score of birth weight was −0.49, which was not so small considering GA. However, it decreased to −2.86 at EC and increased to −1.52 at OPD. Long-term follow-up anthropometric results of ELBW infants excluding those with NEC, syndromes, or disabilities were published in 2017^20^. The z-score of Bwt was −0.88 at birth, −2.16 at 40 weeks of corrected age, and −1.29 at 4 years of age. A direct comparison with our study was difficult, but the z-score of Bwt for our patients was higher at birth and slightly lower at similar follow-up times. There might be a growth retardation in relation to acute abdomen or EF. The difference in z-score of Bwt between the pre-closure and follow-up times was statistically significant in our study (Table 7).

**Table 7:** Growth difference after enterostomy closure.

	Pre-closure	Follow up	*p*-value
Mean z-score	−2.850	−1.549	<0.001
<5 percentile	45	25	0.015
>5 percentile	10	30	

The C/S rate (performed for maternal or female indications) was nearly twice as high as that of vaginal delivery. Although there is inconsistent conclusion whether delivery method is a risk factor for NEC or SIP^21,22^, several articles reported that delivery method affects the intestinal microbiome; thus, it may be a factor in the development of NEC^23,24^. The numbers of singleton and multiple pregnancies were similar between groups. The proportion of ELBW infants in multiple pregnancies was high considering the fact that only 3.9% of all newborns were born as multiples according to a 2016 survey released by Statistics Korea.

Fifteen patients underwent more than two operations before EC: seven due to stenosis or perforation, three after primary closure or anastomosis due to perforation, one who underwent jejunostomy and then ileostomy, one for a perforation during loopogram, one for a second-look operation, one for a for definite operation after a drainage procedure, and one for intestinal biopsy due to a consistently delayed evacuation. Ganglion cells were present at the sigmoid colon and appendix. Eighteen months after biopsy, EC was performed safely and there were no significant evacuation delays. Sixteen patients (29.1%) had enterostomy-related problems at EC: six with high output enterostomy, three with enterostomy prolapse, three with failure-to-thrive, one with high output and prolapse, one with skin problems around the enterostomy, one at parent request, and one due to an accident. One patient had combined high output and enterostomy prolapse. The accidental case consisted of bowel perforation during the loopogram and was closed immediately. One emergency EC was performed due to rapidly progressing enterostomy prolapse.

Complications occurred in 14 of 55 patients (25.5%). The incidence of postoperative complications after EC in infants was reportedly 24–68%^25,26^. All three patients with incisional hernia underwent closure operations. The postoperative ileus patient who underwent reoperation was the same patient above who underwent an emergent operation due to perforation during the loopogram. The second reoperation patient with ileus vomited 5 weeks after EC. The patient did not improve with 10 days of conservative care, so the reoperation was performed. Two patients died during follow-up of causes unrelated to surgery. One patient was discharged due to viral pneumonia and died 2 weeks later due to ongoing dyspnea. One patient died of idiopathic dyspnea.

The MPA used in this study to determine the cut-off value differs from the receiver operating characteristic (ROC) curve, which examines test accuracy based on sensitivity and specificity. MPA considers the number of data differing from ROC. It also implies *p*-value significance, which is used to identify the optimal cut-off value for predictive factors. One study reported that MPA was a stronger predictor than ROC based on a logistic regression analysis in 2016 for predicting the occurrence of major adverse cardiac events^27^. In our study, MPA was used to identify the significant factors for complications and the relevant cut-off values. We found no significant cut-off values for age, corrected age, or enterostomy duration. We found significance only in Bwt at EC, and the most significant value was 2100 g. This value was still significant after adjusting Hb and albumin level. Enterostomy-related problem at EC was highly associated with body weight at EC because of tendency of early EC to solve the problem although not reaching the relevant clinical status for EC. This factor was excluded for multivariable analysis for potential multicollinearity.

Although not statistically significant, median GA was 11 days lower in group 2 than in group 1. Birth weight was nearly same between the two groups, so more patients were small for gestational age in group 1 (50%) than in group 2 (29.7%). Groups were divided according to Bwt at EC, so it is natural that most EC-related characteristics were significantly different. Hb and albumin levels differed between the two groups. This finding implied that group 1 patients not only had a lower median Bwt, they had nutrition-related problems. This speculation is supported by the fact that there is a significant Bwt z-score difference at EC but not at birth. Another significant different factor was enterostomy-related problems at EC: Group 1 had more enterostomy-related problem, and 10 of 16 patients with this problem were diagnosed with high output or failure-to-thrive. This may lead to nutrition differences between the two groups. This study analyzed complications to determine the optimal timing and found that other surgical outcomes were also significant. In group 1, operation time was longer, transfusion within 3 days after EC was more common, mechanical ventilator period was longer, PN period was longer, full feeding day was longer, and hospitalization was longer.

The results of the previous studies on EC timing after acute abdomen in infants can be divided into four categories: 1) early closure is needed for some patients; 2) early closure yields fine results; 3) early closure yields bad results compared to late closure; and 4) no significant difference between early and late closure. Rothstein emphasized the need for early closure in patients with diarrhea and dehydration after EF in 1982^28^. In 1987, the results of early closure were reportedly acceptable in some studies^11,29,30^. However, those studies did not compare early and late closure, nor did they use proper statistical methods. One study of early closure would not be considered early closure in the current study due to differences in definition. Since 2009, one paper each on categories 3) and 4) above has been published. This study yielded results similar to those previously published, i.e. complications and other outcomes were significantly higher in the early versus late closure groups. However, this study is different from the previous studies in that we did not compare the two groups according to values based on our own discretion. This study is the first to find the cut-off value of early and late closure with respect to complications rather than just comparing the two groups. Other outcomes were also significant, showing that the cut-off value was well-determined. One study also suggested the cut-off value for the optimal timing of EC, but the study population and methods were different from our study with significantly different value, 2660 g^7^.

The main limitation of this study is that it was retrospective and included a small number of patients. Although we performed multivariable analysis on the result of MPA method, there may be still confounding factors affecting the occurrence of complications after EC. Further prospective randomized studies that control for patient characteristics are needed.

Body weight at EC was significant factor for complications in ELBW patients. The determined cut-off value was 2100 g. Other outcomes like operation time, hospital stay, mechanical ventilator period, and PN duration were also lower when EC was performed after the patient reached a Bwt of 2100 g at EC.
